# Measuring Motor Competence in Mid-Adulthood: A Reliable Holistic Test (HOLMOT) Sensitive to Sex and Age Differences

**DOI:** 10.3390/jfmk11010104

**Published:** 2026-02-28

**Authors:** José Carlos Cabrera Linares, Pedro Ángel Latorre Román, Juan Antonio Párraga Montilla

**Affiliations:** Department of Musical, Plastic and Corporal Expression, University of Jaén, 23071 Jaén, Spain; jccabrer@ujaen.es (J.C.C.L.); jparraga@ujaen.es (J.A.P.M.)

**Keywords:** motor competence, holistic assessment, mid-adulthood, test–retest reliability, psychometric properties

## Abstract

**Objective**: This study aimed to (i) examine the test–retest reliability of a holistic motor competence test (HOLMOT) in adults aged 30–60 years, and (ii) evaluate its ability to discriminate performance according to sex and age group. **Methods**: A total of 435 adults (206 women and 229 men; Mean age: 43.49 ± 7.55 years; Weight: 69.10 ± 9.88 kg; BMI: 23.81 ± 2.42 kg/m^2^) participated in this cross-sectional study. Motor Competence was assessed using the HOLMOT, a time-based protocol integrating motor-cognitive, locomotor, and manipulative domains. Test–retest reliability was examined in a subsample of 217 participants over a one-week interval using relative (ICC, Pearson’s r) and absolute (SEM, MDC) reliability indices. Sex and age-group differences were analyzed using independent t-tests and analysis of variance. **Results**: The HOLMOT demonstrated good to excellent reliability for the motor-cognitive (ICC = 0.89), locomotor (ICC = 0.94), and total time (ICC = 0.84) outcomes, with low SEM and MDC values. Reliability was lower for the manipulative section (ICC = 0.44). Men exhibited shorter completion times than women across all sections (*p* < 0.001), and adults over 50 years showed significantly longer times in the motor-cognitive and locomotor domains (*p* < 0.05). **Conclusions**: The HOLMOT is a feasible and reliable tool for assessing motor competence in mid-adulthood, demonstrating sensitivity to sex- and age-related differences and supporting holistic, lifespan-oriented models of motor competence.

## 1. Introduction

Motor competence (MC) refers to an individual’s ability to perform a wide range of goal-directed movements in a coordinated, efficient, and adaptable manner, enabling effective interaction with the physical and social environment [1]. Contemporary perspectives conceptualize MC as a multidimensional construct that extends beyond biomechanical proficiency to include perceptual, cognitive, and psychosocial components that collectively shape an individual’s interaction with the physical and social environment [2,3]. This broadened view has important implications for assessment, as instruments focused on isolated outcomes may fail to capture the functional and ecological complexity of motor behaviour in everyday life and sport, underscoring the importance of ecologically grounded approaches to motor competence evaluation [4].

A central theoretical framework in MC research is the developmental model proposed by Stodden and colleagues [5], which describes a dynamic, reciprocal relationship between MC and physical activity (PA) across the lifespan. According to this model, higher MC facilitates engagement in a wider range of physical activities, reinforcing fitness and further motor development, whereas lower MC may constrain participation and promote negative health trajectories [5]. Scientific evidence supports several elements of this framework, indicating that motor skill proficiency in earlier developmental stages predicts subsequent PA and fitness, and that associations among MC, perceived competence, and activity tend to strengthen with age in many cohorts [6,7]. Collectively, these findings highlight MC as a meaningful and actionable target for public health and educational interventions extending well beyond childhood.

Traditionally, research on MC has focused predominantly on childhood and adolescence, documenting strong associations between fundamental motor skills and later physical fitness, psychosocial outcomes, and habitual PA [8,9]. Adolescence, in particular, has been identified as a critical period during which motor trajectories consolidate and begin to track into adulthood [10]. MC remains relevant in adulthood, where movement performance reflects the integration of perceptual-cognitive processes, coordination, and neuromuscular control within context-specific demands. However, fewer studies have examined MC specifically during mid-adulthood (30–60 years). In this stage of life, MC should not be interpreted merely as an indicator of physical fitness or agility. Whereas physical fitness reflects physiological capacities and agility refers to rapid whole-body movement, MC involves the coordinated and adaptive organization of movement solutions to meet complex tasks and environmental demands. From a lifespan perspective, MC, therefore, represents a multidimensional construct supporting efficient and adaptable motor behaviour in everyday and sport-related contexts [11].

Furthermore, distinguishing MC from related constructs is particularly important in adult populations. Coordination refers to the regulation of movement patterns, agility emphasizes rapid changes in direction, and functional ability reflects the capacity to perform activities of daily living. In contrast, MC represents the adaptive integration of these elements, enabling individuals to select, organize, and execute effective movement strategies in dynamic environments. This distinction is especially relevant when assessing relatively healthy adults, whose functional independence may remain intact despite meaningful variability in movement efficiency and motor adaptability [12].

Addressing this gap is warranted, as adulthood is often marked by lifestyle transitions, such as increased work demands and family responsibilities, that frequently coincide with declines in structured PA and increases in sedentary behaviour [13]. These changes may influence motor capacity, functional autonomy, and long-term health. Consequently, expanding MC research into adult populations is necessary to determine whether the relationships observed in youth persist, diminish, or take on new forms across the adult life course [13].

Emerging studies that include wider age ranges suggest that MC remains variable and developmentally meaningful throughout adulthood. Cross-sectional evidence demonstrates that MC assessments can discriminate age-related differences in coordination and task performance, while longitudinal research indicates that adults who sustain regular sport or PA engagement tend to maintain higher levels of MC and fitness into midlife [6,14]. Taken together, these studies suggest that mid-adulthood is not a period of developmental stagnation; instead, MC during this phase appears to reflect a dynamic interplay among prior motor experiences, current activity behaviours, and age-related physiological changes [15].

Sex and age-group differences in MC are well documented in younger populations and often persist into adulthood, although their magnitude and expression vary across domains and measurement approaches. During childhood and adolescence, males typically demonstrate advantages in object-control and power-oriented tasks, whereas females often perform better in balance and fine-coordination tasks. These patterns are shaped by a combination of biological factors, psychosocial influences, and environmental opportunities for practice [16]. In adulthood, some studies continue to observe sex differences in specific motor domains, while others report a reduction in these disparities, suggesting that their persistence depends on lifelong activity behaviours, occupational demands, and broader sociocultural contexts [17,18]. Examining sex- and age-related effects during adulthood is, therefore, essential to determine whether such group differences reflect long-standing developmental divergence or are shaped by modifiable lifestyle and contextual factors.

Nevertheless, methodological limitations complicate the assessment of MC beyond childhood. Widely used test batteries, such as the Test of Gross Motor Development (TGMD), the Movement Assessment Battery for Children (MACB), and the Körperkoordinations test für Kinder (KTK), were primarily developed for children and adolescents and typically rely on either process-oriented qualitative evaluations or isolated product-based outcomes, which may not fully capture the complexity of motor performance in adult populations [2,9]. In contrast, assessment tools designed for older adults generally focus on basic mobility, balance, or fall risk, offering limited sensitivity to differences in coordination and movement efficiency among relatively active adults [19].

This dichotomous approach between process- and product-oriented assessments poses particular challenges in adulthood, where motor performance reflects the integration of coordination, strength, balance, and perceptual-cognitive demands. While process-based measures provide detailed information on movement quality, their feasibility is limited in applied settings, whereas product-based measures may overlook qualitative aspects if considered in isolation [10]. Consequently, there is a clear methodological gap for ecologically valid assessment tools capable of integrating process- and product-related components and detecting subtle performance differences with robust psychometric properties in adult populations.

Holistic assessments frequently employ time-based performance metrics because completion time reflects movement efficiency rather than speed alone. In complex motor tasks that integrate coordination, balance, object control, and decision-making, shorter execution times typically indicate more efficient motor planning, inter-limb coordination, and adaptive control. Therefore, when tasks require accurate execution and rule compliance, total performance time can serve as an ecologically valid indicator of integrated motor competence rather than a simple measure of movement speed [20].

Although time-based performance provides an ecologically valid indicator of integrated motor behaviour, it may also be influenced by physical capacities such as speed, strength, and overall fitness. Therefore, total completion time should not be interpreted as a pure measure of MC. Rather, performance in the HOLMOT emerges from the interaction of coordinative control, perceptual-cognitive processing, balance, and task organization under accuracy and rule constraints, which reduce the likelihood that outcomes are determined solely by speed. Consequently, the test is best interpreted as an indicator of movement efficiency under integrated motor demands.

To address these limitations, holistic approaches to MC assessment have been proposed and recently implemented. Such holistic tests integrate locomotor, manipulative, and perceptual-cognitive components within a single, standardized protocol designed to reflect the complexity of everyday and sport-related motor demands while remaining feasible for field-based administration [11]. In this sense, recent validation studies of a new holistic MC test have shown strong test–retest reliability and discriminative validity supporting its adaptability across different life stages [21]. In this context, a holistic approach does not necessarily imply qualitative movement analysis; rather, it refers to the integrated assessment of multiple motor domains within a continuous task structure that reflects real-world motor demands [11]. Unlike test batteries composed of isolated tasks, holistic assessments require individuals to coordinate perceptual-cognitive processing, locomotor control, and object manipulation within a continuous adaptive performance sequence. Such integration reflects the functional organization of motor behaviour in everyday and sport-related environments and the demands of real-world performance contexts [22].

These findings indicate that a single, well-constructed holistic test can capture key dimensions of MC from youth to late adulthood, provided that task demands, scoring criteria, and normative expectations are appropriately calibrated for the target population. Therefore, the present study aimed to (i) examine the test–retest reliability of a holistic MC test (HOLMOT) in adults aged 30–60 years, and (ii) evaluate its ability to discriminate performance according to sex and age group.

## 2. Materials and Methods

### 2.1. Participants

A total of 435 adults (206 women; mean age = 43.49 ± 7.55 years) participated in this cross-sectional study. Participants were classified into three age groups: 30–39 years (n = 137; 57 men, 41.6%), 40–49 years (n = 167; 106 men, 63.5%), and >50 years (n = 131; 66 men, 50.4%). A priori power analyses were conducted using G*Power 3.1. For the primary age-group comparisons (one-way analysis of variance, three groups), assuming a medium effect size (f = 0.25), α = 0.05, and statistical power of 0.80, a minimum sample size of n = 158 participants (approximately 53 per group) was required. For sex comparisons (independent-samples t-test), assuming a moderate-to-large effect size (d = 0.70), a minimum of n = 68 participants (34 per sex) was required. For the assessment of test–retest reliability, assuming an expected correlation of r = 0.70, a minimum sample of n = 14 participants was required. The final sample size exceeded all minimum requirements. Test–retest reliability was examined in a subsample of 217 participants.

Participants were recruited from several cities located in southern Spain (Andalusia). Inclusion criteria were: (a) aged between 30 and 60 years; (b) absence of physical (e.g., motor), sensory (e.g., visual, auditory, or language), intellectual, or mental disabilities; and (c) provision of written informed consent prior to participation. The study was conducted in accordance with the ethical principles of the Declaration of Helsinki (2013) and received approval from the institutional ethics committee (details omitted to preserve anonymity).

### 2.2. Material and Testing

*Anthropometric measurements*

Body mass was measured using a calibrated digital scale (Seca 899, Hamburg, Germany), and stature was assessed with a portable stadiometer (Seca 222, Hamburg, Germany). Body mass index (BMI) was calculated as body mass (kg) divided by height squared. All measurements were performed by trained evaluators following standardized procedures.

*Design and content validity of the Holistic Motor Competence Test (HOLMOT)*

The development of the HOLMOT involved a panel of five experts in sport sciences who identified and selected motor tasks through a Delphi consensus process. Task selection was guided by established theoretical frameworks of gross MC, integrating locomotor and manipulative skill domains as described in the previous literature. Expert agreement regarding congruence, pertinence, and relevance of the test structure was subsequently evaluated using a Likert-type scale.

*Holistic Motor Competence Test (HOLMOT)*

**Figure 1:**
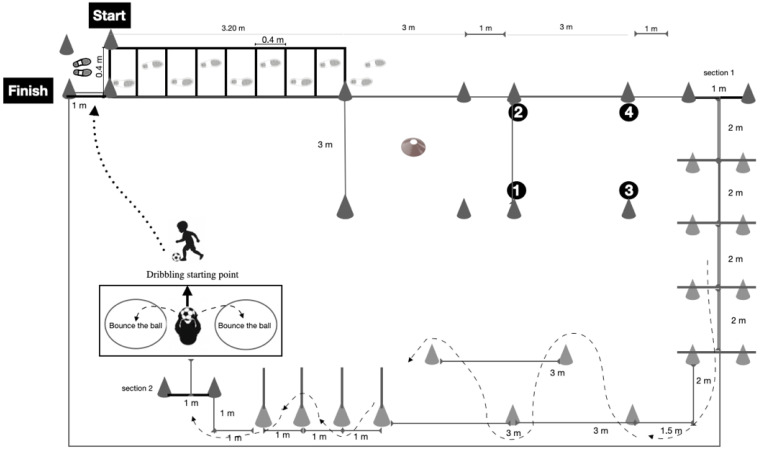
Holistic Motor Competence Test (HOLMOT).

The HOLMOT was administered within a 13 m × 15 m testing area and consisted of three sequential sections, each designed to assess a distinct domain of MC. Total performance time was calculated as the sum of the completion times of the three sections, and individual section times were also recorded. Section 1 assessed motor-cognitive abilities, Section 2 evaluated locomotor abilities, and Section 3 examined manipulative skills involving coordinated use of the upper and lower limbs (Figure 1). The following paragraphs describe each section in more detail.

*Section 1: Motor-cognitive section*

Participants began at a designated starting line and were instructed to run through an agility ladder consisting of eight consecutive squares (40 cm × 40 cm), stepping alternately with the left and right foot. Upon completion, participants entered a 3 m × 3 m area delimited by four large outer cones of different colours (white, yellow, red, and green). Four smaller cones of corresponding colours were positioned at the centre of the area. Participants were required to retrieve each small cone individually and place it onto the matching outer cone, requiring fine motor coordination and colour-based decision-making.

Participants then moved to a second 3 m × 3 m area positioned parallel to the first, where the numbers 1 to 4 were displayed at the corners. They were instructed to step on the numbers in ascending order. To minimize memorization effects, the evaluator rearranged the intermediate numbers before each attempt, while the first and final numbers remained fixed to ensure consistency across trials. After completing this sequence, participants proceeded directly to the next section.

*Section 2: Locomotor section*

The locomotor section required participants to jump consecutively over four low hurdles (20 cm height), spaced two metres apart. Immediately afterward, participants completed a zigzag course defined by five cones within a 6 m × 3 m area, followed by a second zigzag pattern using cones equipped with vertical poles (1 m height) placed at one-metre intervals. Participants were instructed to weave through the course without contacting the cones or poles before proceeding to the final section.

*Section 3: Manipulative section*

The manipulative section assessed object-control and coordination skills. Participants picked up a volleyball and threw it alternately toward two hoops positioned on the ground to their left and right sides. Upon completing the throws, participants placed the ball on the floor and dribbled it using their feet toward the finish line. An attempt was considered valid only when both the participant and the ball crossed the line, at which point timing ceased and section and total completion times were recorded.

### 2.3. Procedure

Prior to testing, the evaluator provided standardized instructions and a demonstration of all test components. Participants completed two familiarization trials to ensure comprehension of the procedures. During testing, participants were instructed to perform the tasks as quickly as possible while maintaining correct execution. Each participant was assessed individually in a separate room to minimize distractions and potential social influence.

Participants completed two official trials, and the best performance was retained for analysis. Any attempt in which one or more sections were performed incorrectly (e.g., touching cones, missing throws, incorrect sequencing) was deemed invalid, and an additional trial was permitted. If an attempt was deemed invalid, an additional trial was allowed until a correct execution was achieved. In all cases, the best valid performance time was retained for analysis. No participant required exclusion due to inability to complete the test correctly. Verbal encouragement was provided in a standardized manner throughout the assessment. All tests were conducted on a level surface under controlled environmental conditions. To evaluate test–retest reliability, the full protocol was re-administered to a subsample of 217 participants one week later under identical conditions.

### 2.4. Statistical Analysis

Content validity was assessed using a Delphi approach involving five experts in sport sciences and PA. Experts rated the congruence, pertinence, and relevance of the test structure using a 5-point Likert scale, and inter-rater agreement was quantified using Fleiss’ kappa coefficient.

Data were analyzed using SPSS Statistics (version 22.0; IBM Corp., Chicago, IL, USA). Descriptive statistics are presented as mean ± standard deviation. Statistical significance was set at *p* < 0.05. Sex differences were examined using independent-samples t-tests. Age-group differences and sex-by-age interactions were examined using two-way analyses of variance (ANOVA). When significant effects were detected, Tukey’s honestly significant difference post hoc tests were applied. Effect sizes for pairwise comparisons were calculated using Cohen’s d.

Test–retest reliability was evaluated using both relative and absolute indices. Relative reliability was assessed using Pearson’s correlation coefficients and intraclass correlation coefficients (ICC) were assessed with 95% confidence intervals, based on a two-way mixed-effects model with absolute agreement. Absolute reliability was examined using the standard error of measurement (SEM) and the minimum detectable change (MDC), expressed as absolute values and percentages. Reliability analyses were additionally performed across sex and age groups to examine measurement stability in demographic subgroups. Paired-samples t-tests and corresponding Cohen’s d values were used to assess systematic differences between test and retest scores.

Agreement between test and retest measurements was further examined using Bland–Altman plots. Associations among the three test sections and total performance time were analyzed using Pearson’s correlation coefficients. Homoscedasticity of test–retest differences was assessed using the Breusch–Pagan test.

## 3. Results

The analysis of content validity yielded a Fleiss’ kappa coefficient of 0.821, indicating a strong level of agreement among the five experts regarding the congruence, pertinence, and relevance of the HOLMOT structure for assessing MC in adults.

Test–retest reliability outcomes are presented in Table 1. No significant differences were observed between test and retest scores for the motor-cognitive section (*p* = 0.806) or for total completion time (*p* = 0.316). Small but statistically significant differences were detected in the locomotor (*p* = 0.019) and manipulative sections (*p* = 0.028); however, the associated effect sizes were trivial (|d| ≤ 0.20). Relative reliability indices demonstrated good to excellent reliability for the motor-cognitive section (ICC = 0.891, 95% CI: 0.850–0.921), the locomotor section (ICC = 0.938, 95% CI: 0.914–0.955), and total time (ICC = 0.837, 95% CI: 0.779–0.881). In contrast, the manipulative section showed lower reliability (ICC = 0.444, 95% CI: 0.299–0.568). Absolute reliability indices supported these findings, with low SEM and MDC values for the motor-cognitive, locomotor, and total time outcomes (SEM ≤ 4.00%; MDC ≤ 11.09%), whereas the manipulative section exhibited substantially higher SEM (19.24%) and MDC (53.32%) values.

Additionally, test–retest reliability of total performance time was further examined across sex and age groups. Reliability remained high in both men (ICC = 0.95) and women (ICC = 0.87). Similarly, ICC values across age groups indicated good to excellent reliability: 0.85 for adults aged 30–39 years, 0.84 for those aged 40–49 years, and 0.99 for participants aged 50 years and older.

**Figure 2:**
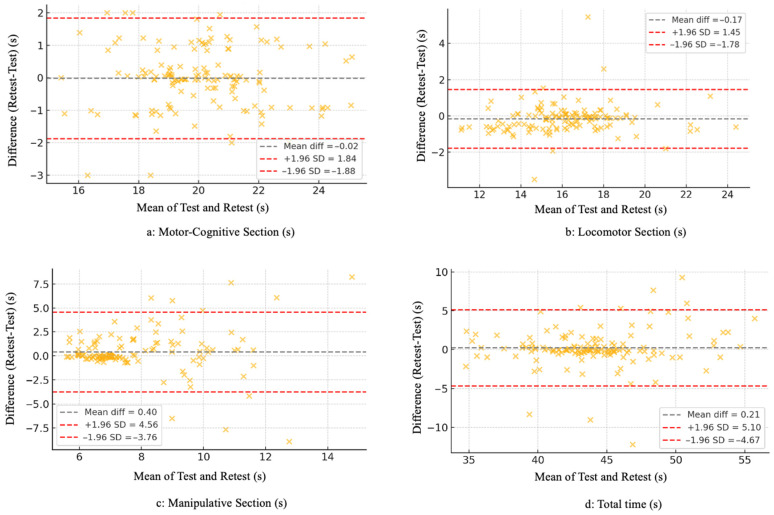
Bland–Altman Plots for Test–Retest Across all Sections of the HOLMOT.

Agreement between test and retest measurements was further examined using Bland–Altman plots (Figure 2). For the motor-cognitive and locomotor sections, test–retest differences were narrowly distributed around zero, with most values falling within the limits of agreement and no evidence of proportional bias. Greater variability was observed for the manipulative section, with a wider dispersion of differences across the measurement range. For total completion time, test–retest differences were small and homogeneously distributed. Homoscedasticity analyses supported constant variance of test–retest differences for the motor-cognitive, locomotor, and total time outcomes (*p* > 0.05), whereas the manipulative section demonstrated heteroscedasticity (*p* < 0.05).

The correlation analysis revealed significant positive associations among all sections of the HOLMOT. The motor-cognitive section showed moderate correlations with both the locomotor (r = 0.323, *p* < 0.001) and manipulative sections (r = 0.406, *p* < 0.001), and a strong correlation with total test time (r = 0.723, *p* < 0.001), indicating that performance in this component substantially contributes to overall completion time. The locomotor section also displayed moderate correlations with the manipulative section (r = 0.393, *p* < 0.001) and a strong association with total time (r = 0.734, *p* < 0.001). These findings suggest that locomotor efficiency is closely linked to overall performance. The strongest relationship emerged between the manipulative section and total test time (r = 0.821, *p* < 0.001), reflecting the substantial influence of this component on the overall duration of the test despite its lower test–retest reliability.

**Figure 3:**
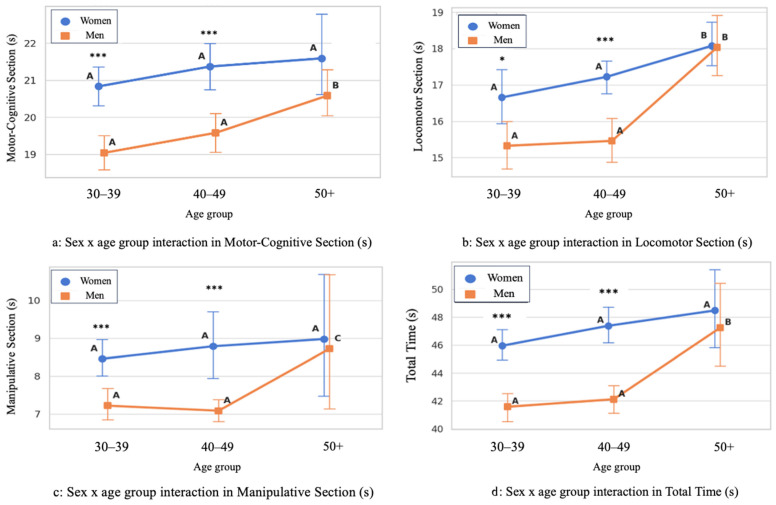
Performance Differences by Sex and Age Group Across HOLMOT Sections and Total Time. Superscript letters (A, B, C) indicate post hoc differences between age groups: * *p* < 0.05; *** *p* < 0.001.

Sex- and age-group differences in performance across the HOLMOT sections are illustrated in Figure 3. Across all sections and age groups, women demonstrated significantly longer completion times than men (*p* < 0.001). Age-group analyses showed a significant increase in completion time with age in both sexes, particularly in participants aged 50 years and older. In this age group, significantly longer times were observed in the motor-cognitive and locomotor sections compared with younger age groups, while total completion time was also significantly higher in the >50 years group (*p* < 0.001).

Anthropometric characteristics and performance outcomes by sex and age group are summarized in Table 2. Men exhibited greater body mass, height, and BMI than women (*p* < 0.05). Regarding age groups, participants older than 50 years presented higher BMI values compared with those aged 30–39 years (*p* = 0.005). This group also showed significantly longer completion times in the motor-cognitive and locomotor sections, as well as in total time, relative to the two younger groups (*p* < 0.05).

## 4. Discussion

The present study aimed to (i) examine the test–retest reliability of a holistic motor competence test (HOLMOT) in adults aged 30–60 years and (ii) evaluate its ability to discriminate performance according to sex and age group. The findings indicate that the HOLMOT demonstrates strong temporal stability in most test sections, effectively differentiates performance by sex and age, and exhibits an internally coherent structure consistent with contemporary multidimensional models of MC. Together, these results extend existing evidence by addressing an important gap in the assessment of MC during mid-adulthood, a life stage for which validated and ecologically grounded instruments remain scarce.

Test–retest analyses revealed good to excellent reliability for the motor-cognitive and locomotor components, whereas the manipulative section demonstrated lower reliability and greater variability. Overall, the HOLMOT demonstrated stronger measurement stability in the motor-cognitive and locomotor domains, whereas the manipulative component showed greater variability and requires further refinement. Furthermore, reliability remained high across sex and age groups, suggesting that the temporal stability of the HOLMOT is consistent across demographic subgroups. These findings suggest a high degree of measurement precision and stability over time, consistent with previous validations of holistic MC assessments in older adult populations [21]. In contrast, the manipulative section showed lower reliability, with reduced ICC values and higher SEM and MDC estimates, indicating greater measurement error. This interpretation is further supported by the greater dispersion observed in the Bland–Altman analysis and the presence of heteroscedasticity in the manipulative section. This increased variability may be related to the higher coordinative and perceptual demands of object-control tasks, which are more sensitive to momentary fluctuations in execution and prior experience. From a task-specific perspective, this section integrates multiple coordinative demands, including rapid ball grasping, alternating throws requiring visuomotor precision under time pressure, and an immediate transition to foot-based ball control. The transition between upper- and lower-limb control strategies may further increase execution variability. In this sense, scientific evidence from perceptually demanding visuomotor tasks in adults suggests that performance precision can vary across testing sessions even in the absence of systematic bias [23]. Also, methodological research indicates that more complex sport-related measures tend to exhibit lower reliability than simpler outcomes [24]. From a methodological perspective, these findings highlight the need for refinement of the manipulative component to enhance the overall psychometric robustness of the HOLMOT. In this sense, the lower reliability observed in this section may be attributed to the greater coordinative complexity and perceptual-motor demands of object-control tasks. Such tasks require precise timing, visuomotor integration, and bilateral coordination, which are more sensitive to moment-to-moment fluctuations and prior experience than locomotor actions. Variability in participants’ previous exposure to ball-handling activities may also have contributed to performance inconsistency between sessions [25,26].

Additional factors may have influenced reliability, including the sequential integration of throwing accuracy and foot-based ball control, the need for rapid transitions between movement patterns, and potential learning effects between test sessions. Although standardized instructions were provided, subtle variations in execution strategy may also contribute to performance variability in complex object-control tasks [27,28].

With respect to sex differences, the HOLMOT consistently differentiated performance between men and women across all test sections, with men demonstrating shorter completion times. These findings are consistent with well-documented sex-related patterns in motor performance across the lifespan, particularly in tasks emphasizing speed, power, and dynamic coordination [18,29]. Such differences may reflect a combination of biological factors, including sex-related differences in neuromuscular characteristics, as well as sociocultural influences such as differential exposure to sport and PA opportunities across development [9,30]. These disparities between sexes can be influenced by a complex interaction between biological factors, such as differences in growth trajectories, neuromuscular development, hormonal regulation, and sociocultural influences, including unequal opportunities for sport engagement and socially constructed expectations regarding PA participation [31].

Age-related analyses demonstrated that adults older than 50 years exhibited significantly longer completion times compared with younger age groups. These findings align with developmental and lifespan frameworks, suggesting that MC remains sensitive to age-related physiological changes, including declines in neuromuscular efficiency, balance control, and cognitive processing speed [1,2]. Moreover, emerging evidence indicates that midlife represents a period during which cumulative lifestyle factors, such as reductions in habitual PA and increasing occupational demands, may begin to manifest in observable changes in motor execution [32,33]. The presence of meaningful variability across adult age groups challenges the assumption that MC remains stable throughout mid-adulthood and supports a dynamic view in which MC reflects the interaction of ageing processes, experiential factors, and ongoing engagement in PA.

From a broader perspective, these findings reinforce the conceptualization of MC as a multidimensional and developmentally relevant construct across the adult lifespan. The observed associations among test sections and total performance time further support the internal coherence of the HOLMOT, suggesting that motor-cognitive, locomotor, and manipulative domains contribute jointly to holistic motor performance. Despite its lower reliability, the strong association between the manipulative section and total performance time suggests that this component captures a meaningful, albeit more variable, aspect of holistic MC. However, the high MDC observed for the manipulative section indicates that substantial performance changes would be required to confirm real individual improvement. Therefore, caution is warranted when using this component to monitor small changes over time in longitudinal or intervention contexts. Future refinements may include additional familiarization trials, modification of task constraints, or adjustments to scoring procedures to reduce variability while preserving ecological validity for adult populations. Despite its lower reliability, the manipulative section demonstrated the strongest association with total performance time. This finding suggests that object-control performance represents a substantial component of overall task completion. Importantly, the higher variability observed in this section likely reflects its sensitivity to individual differences in coordinative proficiency and task familiarity rather than random measurement error. Therefore, while the section introduces greater variability, it also captures a meaningful dimension of holistic MC. This integrated approach is in line with recent perspectives that support ecologically valid assessments designed to capture the multifaceted nature of real-world motor demands [11].

From an assessment perspective, retaining the manipulative component within the total score presents both challenges and advantages. While its lower reliability suggests caution when interpreting small performance changes, its strong contribution to total performance indicates that it captures a relevant aspect of MC that would be overlooked if excluded. Consequently, the total HOLMOT score should be interpreted as an integrated performance index, with attention to section-specific variability when monitoring change over time. Accordingly, the manipulative component should be considered a core domain of holistic MC, although further refinement is warranted to improve measurement stability.

### Limitations and Strength

Several limitations should be acknowledged. First, the cross-sectional design used to examine sex and age-group differences limits causal inference. Although test–retest reliability was assessed longitudinally, only a single follow-up interval was included. Second, the lower reliability observed in the manipulative section suggests that task content or scoring criteria may require further refinement for adult populations. Future refinements may include increasing standardization of task execution, the provision of additional familiarization trials, and adjusting task constraints to reduce variability while preserving ecological validity. Third, relevant factors such as habitual PA levels, physical fitness, and executive function were not assessed and may have contributed to between-individual variability in performance.

Despite these limitations, the study has notable strengths. The use of a holistic, ecologically grounded MC assessment represents a key methodological advantage, as the protocol integrates perceptual, locomotor, and manipulative demands within a single, field-based test. The large sample size, broad adult age range, and comprehensive evaluation of multiple motor domains further enhance the robustness and applicability of the findings. Collectively, these strengths support the utility of the HOLMOT as a feasible assessment tool for examining MC across mid-adulthood.

## 5. Conclusions

In conclusion, the HOLMOT appears to be a feasible and psychometrically sound tool for assessing MC in adults aged 30–60 years. The test is able to discriminate performance by sex and age and to capture both general and domain-specific dimensions of MC, while demonstrating strong reliability for the motor-cognitive and locomotor components and for overall test performance. Although further refinement of the manipulative section is warranted due to its lower reliability, this component shows a strong association with total performance time, suggesting that it captures a relevant, albeit more variable, aspect of holistic MC. Importantly, the HOLMOT is a low-cost, easy-to-administer assessment that does not require specialized equipment or highly specialized personnel beyond professionals trained in sport and exercise sciences, supporting its potential application in epidemiological research, applied settings, and longitudinal monitoring of MC across adulthood.

## Figures and Tables

**Table 1:** Test–Retest Reliability of the Holistic Motor Competence Assessment.

Variable	Test	Re-Test	*p*-Value	R	R^2^	ICC (95% Confidence Interval)	SEM (%)	MDC (%)	Effect Size (Cohen’s d)
Motor-cognitive section (s)	20.11 (2.05)	20.09 (2.00)	0.806	0.890	0.793	0.891 (0.850–0.921) ***	0.67 (3.33%)	1.86 (9.24%)	−0.020
Locomotor Section (s)	16.24 (2.33)	16.08 (2.42)	0.019	0.940	0.884	0.938 (0.914–0.955) ***	0.58 (3.61%)	1.62 (10.00%)	−0.200
Manipulative section (s)	7.60 (1.97)	8.00 (2.09)	0.028	0.456	0.208	0.444 (0.299–0.568) ***	1.50 (19.24%)	4.16 (53.32%)	0.190
Total time (s)	43.95 (4.18)	44.16 (4.54)	0.316	0.840	0.705	0.837 (0.779–0.881) ***	1.76 (4.00%)	4.89 (11.09%)	0.090

SEM: Standard error of measurement; MDC: Minimum detectable change; *** *p* < 0.001.

**Table 2:** Anthropometric and Holistic Test Results Separated by Sex and Age Group.

Variable	Total Mean (SD) n = 435	Men Mean (SD) n = 229	Women Mean (SD) n = 206	*p*-Value	Effect Size (Cohen’s d)	Group 1 (30–39 Years) n = 137	Group 2 (40–49 Years) n = 167	Group 3 (>50 Years) n = 131	*p*-Value	Effect Size (Cohen’s d)	Post Hoc
Age (years)	43.49 (7.55)	43.91 (7.76)	43.07 (7.34)	0.391	0.112	35.62 (2.42) a	44.51 (2.88) b	54.23 (3.34) c	<0.001	4.377	a < b; a < c; b < c *
Weight (kg)	69.10 (9.88)	73.83 (9.13)	64.50 (8.29)	<0.001	1.07	67.36 (10.22)	70.48 (9.90)	69.36 (8.97)	0.101	0.21	
Height (m)	1.70 (0.07)	1.74 (0.06)	1.65 (0.05)	<0.001	1.431	1.70 (0.09)	1.70 (0.06)	1.69 (0.05)	0.874	0.06	
BMI (kg/m^2^)	23.81 (2.42)	24.20 (2.23)	23.44 (2.54)	0.015	0.319	23.14 (2.21) a	24.27 (2.54) b	24.04 (2.31) a	0.005	0.321	a < b *
Motor-Cognitive Section (s)	20.43 (2.20)	19.63 (1.85)	21.21 (2.25)	<0.001	0.762	20.03 (1.85) a	20.43 (2.31) a	21.08 (2.40) b	0.024	0.322	a < b *
Locomotor Section (s)	16.61 (2.36)	16.01 (2.47)	17.19 (2.10)	<0.001	0.512	16.06 (2.47) a	16.30 (2.15) a	18.05 (1.96) b	<0.001	0.605	a < b *
Manipulative Section (s)	8.11 (2.85)	7.51 (2.60)	8.70 (2.96)	<0.001	0.425	7.91 (1.69)	7.89 (2.39)	8.85 (4.54)	0.102	0.199	
Total Time (s)	45.12 (5.69)	43.14 (5.49)	47.05 (5.22)	<0.001	0.731	44.00 (4.27) a	44.62 (4.88) a	47.84 (7.90) b	<0.001	0.437	a < b *

BMI: Body mass index; Superscript letters (a, b, c) indicate post hoc differences between age groups: * *p* < 0.05.

## Data Availability

The data that support the findings of this study are available from the corresponding author upon reasonable request.

## Abbreviations

The following abbreviations are used in this manuscript:

BMI	Body Mass Index
HOLMOT	Holistic Motor Competence Test
MC	Motor Competence
MDC	Minimum Detectable Change
PA	Physical Activity
SEM	Standard Error of Measurement
